# Central Mucoepidermoid Carcinoma Radiographically Mimicking an Odontogenic Lesion

**DOI:** 10.1155/2023/5714099

**Published:** 2023-09-27

**Authors:** Lucas Novaes Teixeira, Erick Gomes Perez, Ana Cláudia Garcia Rosa, Sandro Régis Rodrigues Lima, Mariana Quirino Silveira Soares, Fabricio Passador-Santos, Vera Cavalcanti de Araújo, Andresa Borges Soares

**Affiliations:** ^1^Faculdade São Leopoldo Mandic, Campinas, SP, Brazil; ^2^Federal University of Tocantins, Palmas, Tocantins, Brazil

## Abstract

Central mucoepidermoid carcinoma (CMEC) is a rare pathological entity with only a few case reports in the literature. The present case reported an uncommon occurrence of CMEC mimicking an odontogenic lesion in a young patient. A 17-year-old female patient sought dental care due to a slight swelling located in the posterior region of the mandible on the left side. Radiographic exams revealed an osteolytic lesion with defined limits in relation to proximity to the pericoronal follicle of tooth #38. The clinical and radiographic diagnostic hypothesis was an odontogenic lesion. Histological sections showed the presence of a neoplasm of glandular origin, not encapsulated, with a predominantly cystic growth pattern. The neoplasm consisted of mucous, intermediate, and squamous cells. In the immunohistochemical staining, the neoplastic cells were positive for cytokeratin 7. Mucous cells were positive for PAS with diastase digestion. The final diagnosis consisted of mucoepidermoid carcinoma. The tumor was removed surgically, and the patient has shown no signs of relapse nor recurrence. In conclusion, CMEC may mimic radiographic features of various pathologies, but despite its rarity, clinicians and oral radiologists should consider CMEC as a diagnostic hypothesis for jaw lesions.

## 1. Introduction

The most frequent malignant salivary gland tumor in both adults and children is mucoepidermoid carcinoma (MEC), which accounts for 5-15% of all salivary gland neoplasms and around 30% of all salivary malignancies [1, 2].

MEC can develop in any salivary gland. The majority of MECs arise in the major salivary glands, with the parotid gland being the predominant site [3]. MECs can also arise in minor salivary glands and ectopic salivary gland tissue, with the minor glands of the palate most commonly affected [4]. These tumors can occasionally develop inside the facial skeleton, when they are known as intraosseous or central mucoepidermoid carcinoma (CMEC) [5–7].

The present report describes the clinical and histopathologic features of an uncommon case of CMEC mimicking an odontogenic lesion in a young patient.

## 2. Case Report

**Figure 1:**
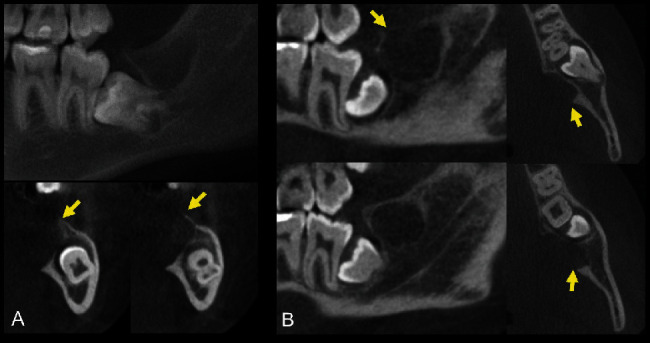
Osteolytic lesion (arrows) with well-defined limits in relation to proximity to the pericoronal follicle of tooth #38. Note the expansion and destruction of the lingual cortical bone of the mandible. (A) Panoramic and parasagittal reconstruction; (B) sagittal and axial reconstructions.

**Figure 2:**
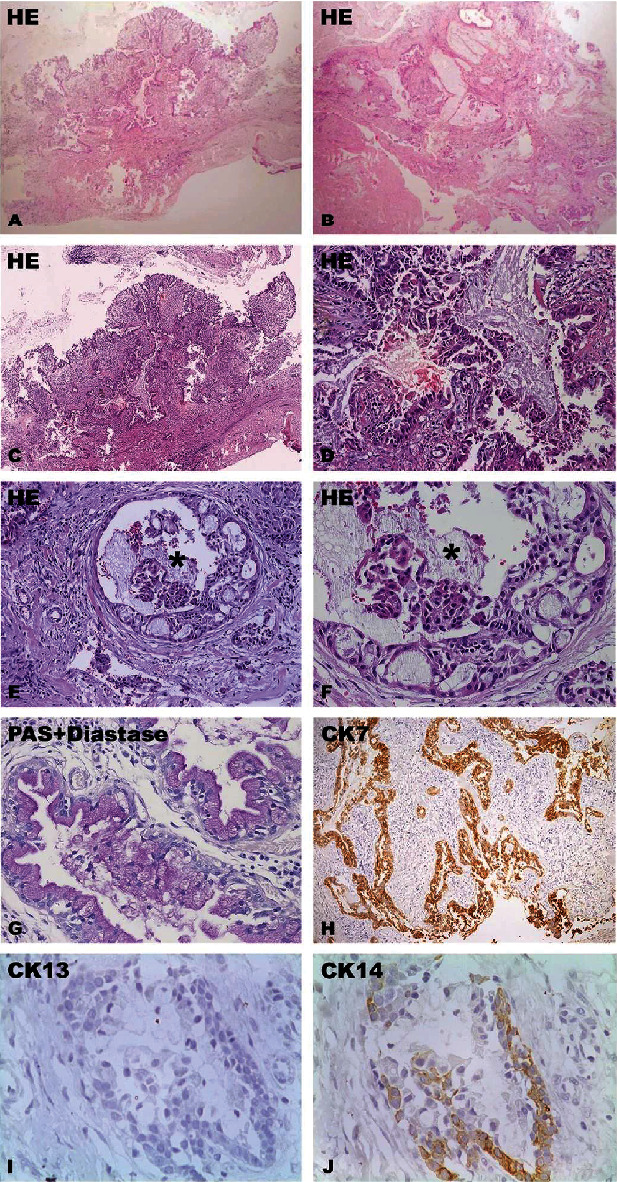
Histological and immunohistochemical features. HE staining revealed a nonencapsulated neoplasm of glandular epithelial origin (A–D). The tumor was comprised with mucous, intermediate, and squamous cells, with cystic growth patterns in some areas (asterisks) (E, F). A large amount of mucus was seen throughout the neoplasm. Mucous cells were positive for PAS with diastase digestion (G). Neoplastic cells were positive for cytokeratin (CK) 7 (H) and CK14 (J) and negative for CK13 (I). Objetives: (A) 1x, (B) 2x, (C) 4x, (E, H) 10x, (F, G) 20x, and (I, J) 40x.

A 17-year-old female patient sought dental care due to a slight swelling located in the posterior region of the mandible on the left side. From imaging examination using cone beam computed tomography, the presence of osteolytic lesion with defined limits in relation to proximity to the pericoronal follicle of tooth #38 were observed. The lesion showed expansion and destruction of the lingual cortical bone of the mandible (Figures 1(A) and 1(B)). From anamnesis, the patient did not report pain and could not specify when the lesion started. An incisional biopsy was performed. During the procedure, the presence of a cystic lesion with liquid and semisolid content was noted. From pathological examination, histological sections revealed the presence of a neoplasm of glandular origin, not encapsulated, with a predominantly cystic growth pattern. The neoplasm consisted of mucous, intermediate, and squamous cells. A large amount of mucous was seen throughout the neoplasm (Figures 2(A)–2(F)). In the histochemical staining with periodic acid–Schiff (PAS) with diastase digestion and mucin staining, the presence of mucous cells was observed (Figure 2(G)). In the immunohistochemical staining, the neoplastic cells were positive for cytokeratin 7 and cytokeratin AE1/AE3. A few neoplastic cells were positive for cytokeratin 14. The cytokeratin 13 and Ki-67 expression was negative (Figures 2(H)–2(J)). The final diagnosis consisted of mucoepidermoid carcinoma. The patient was referred to the head and neck surgeon, and the tumor was removed surgically. Four years later, the patient is still being monitored and shows no signs of relapse or recurrence.

## 3. Discussion

CMEC is a very rare malignant tumor of the salivary gland and comprises 2-4% of all MEC [8]. The present report showed a rare case of CMEC diagnosed in a 17-year-old female, with clinical and radiography features mimicking an odontogenic lesion.

The origin and pathogenesis of CMEC still remain very controversial. Several speculations have been described, and among them, the most reported are mucous metaplasia and neoplastic transformation of the epithelium of an odontogenic cyst; entrapment of the submandibular, sublingual, or other minor glands within the mandible during embryonic development, which subsequently undergo neoplastic transformation; neoplastic transformation of maxillary sinus epithelium; and remnants of the dental lamina. The most likely source of central MEC is the neoplastic transformation of the epithelial lining of an odontogenic cyst, since mucus-producing cells are commonly found in odontogenic cyst linings [9–12]. The origin of CMEC in our case could not be determined; radiographically, the tumor was in proximity to the pericoronal follicle of an impacted tooth, but histologically, there was no evidence of any odontogenic epithelium.

The literature proposed six diagnostic criteria to establish the CMEC diagnosis: presence of an intact cortical plate, presence of a radiographic distinct osteolytic lesion, positive mucin staining, absence of primary lesion in the salivary gland, exclusion of an odontogenic tumor or metastasis, and histological confirmation [10, 13, 14]. The present case meets almost all the diagnostic criteria for CMEC, except for the presence of an intact cortical plate. In the present case, the lesion showed expansion and destruction of the lingual cortical bone of the mandible. This diagnostic criterion was first proposed by Silverglade et al. [15] and has been widely used. The authors have cataloged a series of CMEC considering (1) the presence of intact cortical plates, (2) radiographic evidence of bone destruction, and (3) histopathologic examination of the lesion as evidence of the central origin of the lesions. However, the presence of intact cortical plates as reported by them was evaluated by means of a conventional radiographic image. Bidimensional imaging presents inherent limitations, such as structure superposition, that could hamper accurate identification of cortical destruction. In fact, other published articles reporting CMEC tomographic images have shown jawbone cortical destruction [8, 16–18]. We suggest that the presence of intact cortical plates could be removed as one of the criteria for the diagnosis of CMEC.

The majority of cases of CMEC have been reported occurring in the mandible [5, 7]. Females were slightly more affected than males (ratio male:female 1 : 1.07), with high incidences in the fifth decade [5, 7]. Zhou et al. described thirty-nine cases of CMEC, with only two cases diagnosed in patients younger than 18 years old (15 and 16 years old). Similar to other case reports in the literature, our case was detected in a female patient, and it was located in the mandible. The lesion, however, developed in a 17-year-old patient, which is an unusual finding. Swelling, a slow-growing lesion, pain, trismus, fistula, and paresthesia are some of the clinical symptoms of CMEC [5]. In the present case, swelling was the only symptom reported by the patient.

Radiographically, CMEC may present a variety of radiographic aspects, such as well or ill-defined periphery, unilocular or multilocular radiolucency. Rarely, there has been a mixed radiolucent-radiopaque feature. Tooth displacement, cortical expansion, and cortical perforation could also be found. These radiographic features are commonly found in odontogenic cysts and tumors [16]. According to Chan et al. [16], oral radiologists should consider CMEC in the radiographic differential diagnosis of multilocular lesions of the jaws if there are two common findings, namely, internal sclerotic bony masses and perforation of the external cortex with extension into surrounding soft tissue. In the literature, most reported cases show radiographically extensive lesions. The present case shows an unusual radiographic appearance. The case presents a relatively small, unilocular, radiolucent lesion with defined limits and in relation to proximity to the pericoronal follicle of an impacted tooth, with radiographic features similar to an odontogenic lesion, as a keratocyst, ameloblastoma, or ameloblastic fibroma.

The histological features of the CMEC are the same as those of MEC, i.e., the tumor is characterized by variable components of epidermoid, mucous, and intermediate cells, with a cystic and solid growth pattern. CMEC could be classified into low, intermediate, or high grade based on certain histological parameters such as cell type component, cystic component, necrosis, anaplasia, mitoses, and neural invasion [4]. Similar to the present case, the majority of CMEC cases are found to be of histologically low grade [5–7].

Low-grade MEC has histological characteristics similar to glandular odontogenic cyst. According to Fowler et al. [19], the glandular odontogenic cyst has the following histological features: eosinophilic cuboidal cells, microcysts, clear (vacuolated) cells, variable thickness, apocrine snouting, mucous cells, epithelial spheres, tufting (papillary projections), multiple compartments, and cilia. The authors suggest that the presence of 7 or more microscopic parameters was highly predictive of a diagnosis of glandular odontogenic cyst. There was no evidence of eosinophilic cuboidal cells, variable thickness, apocrine snouting, epithelial spheres, tufting (papillary projections), or cilia in the current case.

Conservative procedures and radical surgery are the possible treatment strategies for CMEC. Neck dissection and adjuvant treatment are still controversial [7]. The prognosis depends on the histological grade. Low-grade histology shows a good prognosis [7]. In the current case, conservative surgery was performed, and the patient has not had a recurrence after 4 years of follow-up.

In conclusion, CMEC is an uncommon pathological entity with only a few case reports in the literature. The current case describes a CMEC mimicking an odontogenic lesion in a young patient. For this reason, despite CMEC rarity, clinicians and oral radiologists should consider CMEC as a diagnostic hypothesis for jaw lesions.
